# Effects of different human body parameters on the vertical dynamic characteristics of a pedestrian structure perception coupled system

**DOI:** 10.1038/s41598-025-95094-8

**Published:** 2025-03-22

**Authors:** Di Liu, Yanyan Zhou, Xinglong Pu, Qiankun Zhu, Binbin Zhang

**Affiliations:** 1 College of Architecture and Civil Engineering, Shangqiu Normal University, Shangqiu, 476000 People’s Republic of China; 2 School of Electronic and Electrical Engineering, Shangqiu Normal University, Shangqiu, 476000 Henan People’s Republic of China; 3 Institute of Earthquake Protection and Disaster Mitigation, Lanzhou University of Technology, Lanzhou, 730050 People’s Republic of China

**Keywords:** Human dynamic parameters, Lightweight floor, Pedestrian-floor-perception coupling equation, Dynamic characteristics, Vibration serviceability, Civil engineering, Mechanical properties

## Abstract

Different dynamic parameters of the human body will affect the vertical dynamic characteristics of the structure. If this parameter is ignored, it will affect the serviceability evaluation results. To consider the influence of different human dynamic parameters on the human-induced vibration of the floor, the pedestrian-structure-perception coupling equation is established in this paper, taking the lightweight CFS composite floor as an example. 60 sitting people of different ages and genders are selected as perceptions, the dynamic parameters of the human body are mass, stiffness, and damping. By calculating the frequency and damping ratio of the floor when pedestrians pass through the floor at an asynchronous frequency, as well as the dynamic response of the floor and the perceiver, The effects of human dynamic parameters on the dynamic characteristics of pedestrian-structure-perception coupling system are compared. The results show that with the change of pedestrian walking position, in the pedestrian-structure-perception coupling system, when the pedestrian walks near the mid-span of the structure, the frequency of the structure reaches the minimum and the damping ratio of the structure reaches the maximum. The influence of men’s dynamic parameters on the frequency and damping of the structure is greater than women and children, with values of 3.74% and 186.50%. Comparison of acceleration 1-s RMS, the perceiver is larger than the floor. When evaluating the serviceability of the floor, the interaction of the pedestrian-structure-perception coupling system should be considered.

## Introduction

With the development of building materials, lightweight and high-strength materials are particularly favored in design and application, which has resulted in more and more lightweight floors and lightweight pedestrian bridges^1,2^. The mass of the lightweight floor is only about half of that of the concrete floor, or even less, which leads to a more significant problem of human induced vibration comfort^3–5^.

There are many literatures on the comfort of human-induced vibration. In the calculation of pedestrian load, some scholars regard the effect of pedestrians on the structure simply as pedestrian load^6–8^, and some scholars study the comfort of human induced vibration by considering the pedestrian structure interaction. For example, Cao et al.^9^ studied the human induced vibration of pedestrian bridges by considering the vertical pedestrian structure interaction. Xie et al.^10^ studied the human structure interaction based on the self excitation manikin, and proposed that the human structure interaction will gradually increase with the increase of the mass ratio of human and structure. Zhu et al.^11^ evaluated the vibration comfort of the fabricated lightweight floor by considering the human structure interaction in the form of probability. Zhang et al.^12^ studied the dynamic response of cold-formed thin-wall steel (CFS) composite floor under pedestrian load by considering the interaction between people and structure. Cássio et al.^13^ studied the dynamic response of the structure using the bio-mechanical model. Hui et al.^14^ assessed the comfort of human induced vibration of a steel structure corridor based on human structure coupling vibration. For the literatures on human induced vibration of structures by human body parameters, there are many literatures on pedestrian bridge^15–18^, while there are few research literatures on floors.

The above literatures have a great contribution on comfort of human-induced vibration. The factors considered in the calculation on the dynamic response of the floor can be divided into three aspects: one is to simply simplify pedestrians as Fourier load models and apply them to the floor in calculation; the other is to simplify pedestrians as single degree of freedom models and consider the pedestrian structure coupling in calculation; the third is that pedestrians on the floor are mostly considered by walking or standing. In the above three aspects, the dynamic response of the perceivers on the floor is first ignored. That is, the dynamic response of the structure under the pedestrian structure coupling system is used to replace the dynamic response of the real comfort perception person under the pedestrian-structure-perception coupling system. Secondly, as the perceivers, their posture and age are different, and their human dynamic parameters are also different. As the perceivers, they have their own responses, and this response is related to the human dynamic parameters of the perceivers. For lightweight floors, human dynamic parameters have a great impact on the response of the floor to human induced vibration. If the impact is ignored for lightweight floors, the evaluation results may be inaccurate.

When evaluating the vibration comfort of real-life engineering structures, the peak acceleration is relatively convenient to apply and intuitive. It assumes that the perceiver is a rigid body rather than a dynamic system with stiffness, damping, and mass. However, the limitation in reality is that when establishing the human vibration comfort evaluation system, the structural force response is often simply used to replace the perception response of the perceiver to vibration. In this paper, when establishing the dynamic model and conducting engineering experimental analysis, it is considered necessary to refer to existing research and take into account the peak acceleration of the perceiver on the structure, instead of just roughly using the structural dynamic response as the evaluation guide.

In order to consider the influence of human dynamic parameters (different mass, stiffness and damping of different ages and genders) on the comfort of light floor induced vibration, this paper establishes the pedestrian-structure-perception coupling equation. Taking light floor as an example, 60 people of different ages and genders are selected as the perceptors. By calculating the frequency and damping ratio of the floor when pedestrians pass through the floor in an asynchronous frequency, and the dynamic response of the floor and the perceivers, the influence of different human dynamic parameters on the dynamic characteristics of the pedestrian-structure-perception coupling system is compared.

## Pedestrian-floor-perceptor coupling governing equation

In the daily use of building structures, when the amplitude of vibration exceeds a certain range, pedestrians on the floor begin to experience discomfort. For example, users on the structure may perceive the vibration, suffer from decreased concentration,and in severe cases, experience physical and psychological discomfort symptoms such as dizziness and palpitations. These symptoms may even lead to public safety incidents caused by panic among the crowd.

When establishing the coupling governing equation, the interaction between pedestrian, structure and perceiver is mainly considered. The floor vibration generated by pedestrian walking may not cause discomfort due to the different dynamic parameters of pedestrians, but may cause discomfort to the perceiver. In this study, the most commonly used office environment seating is selected as the research object, which is a limitation of the study. Within this research object, there are primarily two forms of occupants: pedestrians and seated individuals.

The full path of excitation-propagation-perception considered in this study excludes external excitation sources of the building structure, such as vehicle traffic and environmental vibrations. This is a limitation in terms of the dynamic excitation sources considered. Only pedestrians are taken as the excitation source, while the floor system serves as the propagation medium and the seated individuals act as the perceivers.

Generally, office spaces are required to maintain a quiet and stable working environment. Therefore, it is essential to prevent excessive floor vibrations caused by human-induced excitations, which could adversely affect the working conditions of the perceivers.

### Establishment of coupling equations

**Fig. 1:**
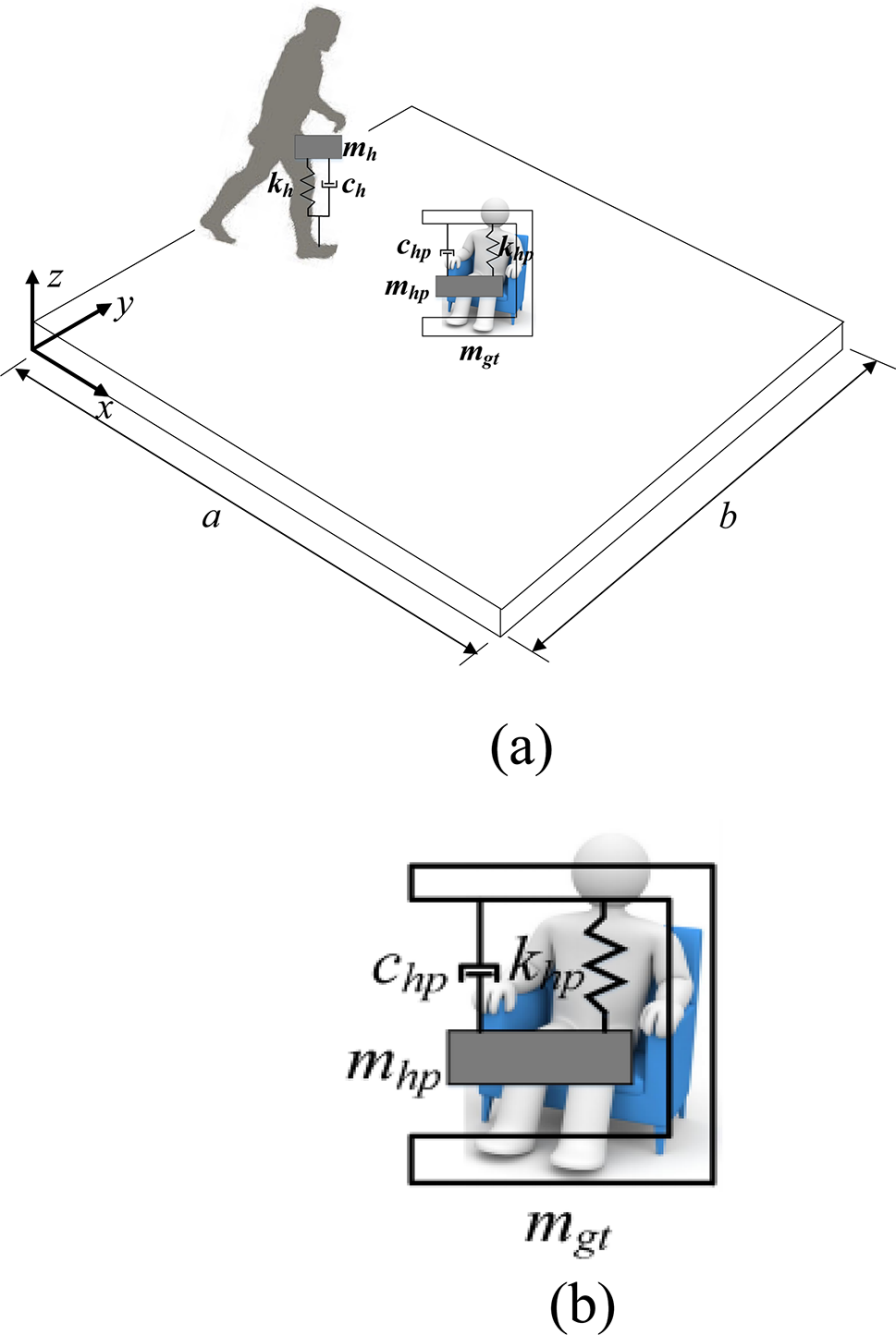
(**a**) Schematic diagram of pedestrian-floor-sensor coupling vibration. (**b**) The single-degree-of-freedom biomechanical model of the perceiver.

Figure 1 is a schematic diagram of pedestrian-floor-sensor coupling vibration. Both the pedestrian and the perceiver are equivalent to a single degree of freedom biomechanical model, $m_{h}$, $c_{h}$ and $k_{h}$ respectively represent the mass, damping and stiffness of the pedestrian^19^. Mass $m_{hp}$ and $m_{gt}$, damping $c_{hp}$ and stiffness $k_{hp}$ are used to represent the human dynamic parameters of the perceiver respectively. The pedestrian-floor-perceiver coupling equation can be established according to the overall dynamic balance.

The research object in this paper encompasses individuals on the floor slab who are either standing or sitting. These individuals themselves form a complete dynamic system and possess their own responses, which are related to their postures, such as lying flat, reclining, standing, and squatting. As shown in Fig. 1b of this paper, the human dynamic parameters of the perceivers on the floor slab, including the masses $m_{hp}$ and $m_{gt}$, damping $c_{hp}$, and stiffness $k_{hp}$, represent the responses of all the aforementioned postures.

Thus, based on the overall dynamic equilibrium, the coupled control equations for the pedestrian-floor-perceiver system can be established. Based on the existing work, this paper considers the pedestrian-structure interaction, and establishes the coupling governing equation of pedestrian-floor-perception interaction:

1 $$\begin{aligned} & \alpha \frac{{\partial^{2} W}}{{\partial t^{2} }} + \beta \frac{\partial W}{{\partial t}} + \frac{{\partial^{4} W}}{{\partial X^{4} }} + 2\lambda^{2} \frac{H}{{D_{x} }}\frac{{\partial^{4} W}}{{\partial X^{2} \partial Y^{2} }} \\ & \quad + \lambda^{4} \frac{{D_{y} }}{{D_{x} }}\frac{{\partial^{4} W}}{{\partial Y^{4} }} = \gamma \lambda \delta (X - X_{F} (t))\delta (Y - Y_{F} (t)) \\ & \quad + \chi \lambda \delta (X - X_{F} (t))\delta (Y - Y_{F} (t))[k_{h} W_{h} \\ & \quad - k_{h} W - c_{h} \frac{\partial W}{{\partial t}} + c_{h} \frac{{\partial W_{h} }}{\partial t}] \\ & \quad + \left\{ \begin{gathered} k_{hp} \left[ {z\left( t \right) - W\left( {X_{hp} ,Y_{hp} ,t} \right)} \right] \hfill \\ + c_{hp} \left[ {\dot{z}\left( t \right) - \dot{W}\left( {X_{hp} ,Y_{hp} ,t} \right)} \right] \hfill \\ + (m_{hp} + m_{gt} )g - m_{gt} \frac{{\partial^{2} W}}{\partial t} \hfill \\ \end{gathered} \right\} \\ & \quad \chi \lambda \delta (X - \xi )\delta (Y - \tau ) \\ \end{aligned}$$

The pedestrian dynamic balance equation can be written as:

2 $$m_{h} \frac{{\partial^{2} W_{h} (t)}}{{\partial t^{2} }} + k_{h} W_{h} (t) + c_{h} \frac{{\partial W_{h} (t)}}{\partial t} - k_{h} W(X,Y,t) - c_{h} \frac{\partial W(X,Y,t)}{{\partial t}} = 0$$

The dynamic balance equation of the perceiver can be written as:

3 $$m_{hp} \ddot{z}\left( t \right) + c_{hp} \dot{z}\left( t \right) + k_{hp} z\left( t \right) = c_{hp} \dot{W}\left( {\xi ,\tau ,t} \right) + k_{hp} W\left( {\xi ,\tau ,t} \right)$$

where $W$ is the dimensionless displacement function of the plate, $W_{{\text{h}}}$ is the displacement function of pedestrians, $z\left( t \right)$ represents the displacement function of the perceivers,$\left( {\xi ,\tau } \right)$ represents the position coordinates of the perceiver on the floor. $\alpha = \frac{{\rho ha^{4} }}{{D_{{\text{x}}} }}$, $\gamma = \frac{{a^{2} }}{{D_{{\text{x}}} }}\left( {m_{{\text{h}}} g + F\left( t \right)} \right)$, $\beta = \frac{{ca^{4} }}{{D_{{\text{x}}} }}$, $F\left( t \right)$ is the pedestrian load, and can be expanded into a Fourier series form^20–22^, $\chi = \frac{{a^{2} }}{{D_{x} }}$, *a* and *b* are the length floor, $X = \frac{x}{a}$, $Y = \frac{y}{b}$ are dimensionless coordinates. $X_{F} (t) = \frac{{v_{x} t}}{a}$, $Y_{F} (t) = \frac{{v_{y} t}}{b}$, $v_{x}$, $v_{y}$ are the pedestrian’s speed in the *x* and *y* directions, respectively. $\lambda = a/b$ are is the aspect ratio, *c* is the viscous damping coefficient of the plate. In addition, $D_{x}$ is the bending stiffness of the plate around the *Y* axis; $D_{y}$ is the bending stiffness of the plate around the *X* axis; $H$ is the effective torsional stiffness. $\delta (X - X_{F} (t))$, $\delta (Y - Y_{F} (t))$ is Dirac function.

### Boundary conditions

In this paper, it is considered that the floor is hinged on four sides, and the boundary conditions are as follows:

Where $X = 0$ and $X = 1$, the displacement and bending moment are zeros, namely:

4a $$W = 0{, }\frac{{\partial^{2} W}}{{\partial X^{2} }} = 0 \,$$

where $Y = 0$ and $Y = 1$, the displacement and bending moment are zeros, namely:

4b $$W = 0{, }\frac{{\partial^{2} W}}{{\partial Y^{2} }} = 0 \,$$

## Discretization and solution of equation

The differential quadrature method (DQ method) is described in literatures^23^, and DQ method and integral quadrature method (IQ method) are combined in literatures^23^. The Eq. (1) is discretized by Dirac function, DQ method and IQ method. The following can be obtained:

5 $$\begin{aligned} & \alpha \frac{{\partial^{2} W(X_{i} ,Y_{j} )}}{{\partial t^{2} }} + \beta \frac{{\partial W(X_{i} ,Y_{j} )}}{\partial t} \\ & \quad + \sum\limits_{i = 1}^{n} {A_{ij}^{(4)} } W(X_{i} ,Y_{j} ) + 2\lambda^{2} \sum\limits_{i = 1}^{n} {\sum\limits_{j = 1}^{m} {A_{ij}^{(2)} } \overline{A}_{ij}^{(2)} } W(X_{i} ,Y_{j} ) \\ & \quad + \lambda^{4} \sum\limits_{j = 1}^{m} {\overline{A}_{ij}^{(4)} } W(X_{i} ,Y_{j} ) \\ & = \left\{ {\begin{array}{*{20}c} 0 & {j \ne q} \\ {\frac{1}{{R_{p} \overline{R}_{q} }}\left( \begin{gathered} \lambda \gamma + \chi \lambda [k_{h} W_{h} + c_{h} \frac{{\partial W_{h} }}{\partial t}] \hfill \\ - \chi \lambda [k_{h} W(X_{q} ,Y) \hfill \\ + c_{h} \frac{{\partial W(X_{q} ,Y)}}{\partial t}] + \hfill \\ \chi \lambda \left\{ \begin{gathered} k_{hp} \left[ {z\left( t \right) - W\left( {\xi ,\tau ,t} \right)} \right] \hfill \\ + c_{hp} \left[ {\dot{z}\left( t \right) - \dot{W}\left( {\xi ,\tau ,t} \right)} \right] \hfill \\ \end{gathered} \right\} \hfill \\ + \chi \lambda [(m_{hp} + m_{gt} )g \hfill \\ - m_{gt} \frac{{\partial^{2} W\left( {\xi ,\tau } \right)}}{\partial t}] \hfill \\ \end{gathered} \right)} & {j = q} \\ \end{array} } \right. \\ & \quad j = 1,2,...,m \, \\ \end{aligned}$$

wherein, $A_{ij}^{(r)}$ and $\overline{A}_{ij}^{(r)}$ are respectively the differential quadrature coefficients of $X$ and $Y$ directions, *i* and *j* are respectively the grid points of the floor, $R_{p}$ is the $X$ direction weight coefficient, and $\overline{R}_{q}$ is the $Y$ direction weight coefficient.

The boundary conditions were discretized^24^ and substituted into the equation to obtain:

6 $$\begin{aligned} & \alpha \frac{{\partial^{2} W(X_{i} ,Y_{j} )}}{{\partial t^{2} }} + \beta \frac{{\partial W(X_{i} ,Y_{j} )}}{\partial t} \\ & \quad + \sum\limits_{i = 1}^{n} {C_{1} \cdot } W(X_{i} ,Y_{j} ) + 2\lambda^{2} \sum\limits_{i = 1}^{n} {\sum\limits_{j = 1}^{m} {C_{2} \cdot } } W(X_{i} ,Y_{j} ) \\ & \quad + \lambda^{4} \sum\limits_{j = 1}^{m} {C_{3} \cdot } W(X_{i} ,Y_{j} ) \\ & = \left\{ {\begin{array}{*{20}c} 0 & {j \ne q} \\ {\frac{1}{{R_{p} \overline{R}_{q} }}\left( \begin{gathered} \lambda \gamma + \chi \lambda [k_{h} W_{h} + c_{h} \frac{{\partial W_{h} }}{\partial t}] - \hfill \\ \chi \lambda [k_{h} W(X_{q} ,Y) + \hfill \\ c_{h} \frac{{\partial W(X_{q} ,Y)}}{\partial t}] + \hfill \\ \chi \lambda \left\{ \begin{gathered} k_{hp} \left[ {z\left( t \right) - W\left( {\xi ,\tau ,t} \right)} \right] \hfill \\ + c_{hp} \left[ {\dot{z}\left( t \right) - \dot{W}\left( {\xi ,\tau ,t} \right)} \right] \hfill \\ \end{gathered} \right\} \hfill \\ + \chi \lambda [(m_{hp} + m_{gt} )g \hfill \\ - m_{gt} \frac{{\partial^{2} W\left( {\xi ,\tau } \right)}}{\partial t}] \hfill \\ \end{gathered} \right)} & {j = q} \\ \end{array} } \right. \\ & \quad j = 1,2,...,m \, \\ \end{aligned}$$

where, $C_{1}$ ~ $C_{{3}}$ is the boundary condition coefficient, see in reference^24^.

Equation (2), (3) and (6) can be combined into the following matrix form:

7 $${\text{M}}\ddot{{\text{u}}}({\text{t}}) + C\dot{{\text{u}}}({\text{t}}) + {\text{Ku}}({\text{t}}) = {\text{F}}$$

where ${\text{M}}$_,_ ${\text{C}}$ and ${\text{K}}$ are respectively the mass matrix, damping matrix and stiffness matrix after the coupling of pedestrians, perceptors and floors, and ${\text{F}}$ are load vectors. ${\ddot{\text{u}}}({\text{t}})$, ${\dot{\text{u}(t)}}$ and ${\text{u(t)}}$ are acceleration, velocity and displacement vectors, respectively. The pedestrian load vector is further refined^23^.

## Example analysis

In this paper, a lightweight CFS composite floor is selected to simulate the office environment. Its span a = 8.40 m, width b = 7.20 m, plate thickness h = 0.1657 m, and total mass of the floor m = 5545.20 kg, $D_{x} = {3}{\text{.12}} \times 10^{6} \;{\text{N}}\;{\text{m}}$, $H = 0$, $D_{y} = {0}.{33} \times 10^{5} \;{\text{N}}\;{\text{m}}$, for the calculation of specific parameters, see reference^25^. The damping ratio of the floor under no-load conditions is 0.02. Walking with different step frequency adopts the slow step frequency parameters given in literature^26^. The percepters selected in this paper sit in the middle of the span of the floor, in which the percepters sit and stand in the middle of the span of the floor successively by one person at a time. Table 1 of reference^27^ gives the dynamic parameters of the single degree of freedom of sitting and standing human body, and the mechanical model is shown in Fig. 1.

**Table 1:** Single-degree-of-freedom model 1b fit to the experiment curves.

Subject	Sex	Age	*K*_1_ (N/m)	*C*_1_ (Ns/m)	*M*_1_ (kg)	*M*_2_ (kg)	Total mass (kg)
1	M	26	34,142	1187	1.3	44.6	45.9
2	M	16	41,151	1122	8.8	35.6	44.4
3	M	39	71,772	1845	21.3	86.7	108.0
4	M	38	62,976	1631	4.3	52.8	57.1
5	M	34	34,653	1312	5.0	47.8	52.8
6	M	33	29,409	675	12.9	31.0	43.9
7	M	29	54,623	1658	11.7	60.5	72.2
8	M	25	35,756	1009	13.0	39.1	52.1
9	M	45	36,286	898	15.3	32.7	48.0
10	M	51	66,748	1705	0.1	65.8	65.9
11	M	16	38,962	985	5.9	35.6	41.5
12	M	27	34,822	954	17.2	39.0	56.2
13	M	56	70,926	1447	6.8	59.0	65.8
14	M	17	54,085	1475	1.4	59.4	60.8
15	M	69	71,813	1173	20.9	34.7	55.6
16	M	27	46,384	1797	2.1	51.3	53.4
17	M	39	66,593	1377	4.7	51.5	56.2
18	M	39	66,803	1833	3.4	80.4	83.8
19	M	50	42,940	1286	12.2	46.7	58.9
20	M	45	77,829	2345	2.1	76.2	78.3
21	M	17	48,025	1165	13.6	46.5	60.1
22	M	23	42,443	1083	3.1	43.9	47.0
23	M	23	52,609	1204	17.3	40.7	58.0
24	M	17	63,948	1636	0.9	43.3	44.2
Mean of 24 men			51,987	1366	8.6	50.2	58.8
25	F	24	26,951	957	4.8	38.9	43.7
26	F	56	48,045	1217	11.7	48.1	59.8
27	F	22	58,890	1486	0.5	45.3	45.8
28	F	45	40,143	1565	3.1	48.6	51.7
29	F	55	58,186	1277	1.5	39.7	41.2
30	F	52	37,755	1792	0.4	52.8	53.2
31	F	25	36,342	1170	12.1	39.9	52.0
32	F	23	38,886	1925	1.1	52.2	53.3
33	F	40	32,252	621	18.2	33.5	51.7
34	F	23	32,174	935	13.8	36.5	50.3
35	F	17	45,515	1403	2.2	50.7	52.9
36	F	35	38,227	1178	15.4	41.2	56.6
37	F	25	43,578	976	16.7	34.5	51.2
38	F	39	35,351	1076	8.2	39.5	47.7
39	F	21	46,037	1577	2.6	53.2	55.8
40	F	38	39,493	823	11.9	28.8	40.7
41	F	24	50,712	1172	9.6	38.4	48.0
42	F	31	30,671	880	17.4	47.1	64.5
43	F	59	52,524	1319	1.6	58.7	60.3
44	F	21	52,151	1003	12.1	31.8	43.9
45	F	41	35,154	826	14.9	37.4	52.3
46	F	38	38,850	1435	0.1	50.2	50.3
47	F	22	39,338	1909	0.8	42.9	43.7
48	F	31	42,586	994	18.8	40.8	59.6
Mean of 24 women			41,659	1230	8.3	42.9	51.3
49	F	10	34,387	421	12.5	19.4	31.9
50	F	11	32,487	762	7.0	26.4	33.4
51	F	7	24,887	511	2.1	21.3	23.4
52	F	9	37,977	923	2.2	31.1	33.3
53	F	11	36,203	992	3.8	30.6	34.4
54	F	14	28,960	734	5.5	24.7	30.2
55	M	11	35,428	753	3.1	28.2	31.3
56	M	13	47,668	1564	0.3	50.9	51.2
57	M	12	25,937	820	11.1	34.9	46.0
58	M	13	31,973	607	14.2	30.9	45.1
59	M	8	33,395	718	3.5	27.5	31.0
60	M	13	31,032	1387	0.3	41.4	41.7
Mean of 12 children			33,361	849	5.5	30.6	36.1
Mean of 60 subjects			44,130	1485	7.8	43.4	51.2
Fit mean of 60 subjects			44,115	1522	4.1	46.7	50.8

### Influence of different human body parameters on structural dynamic parameters

**Fig. 2:**
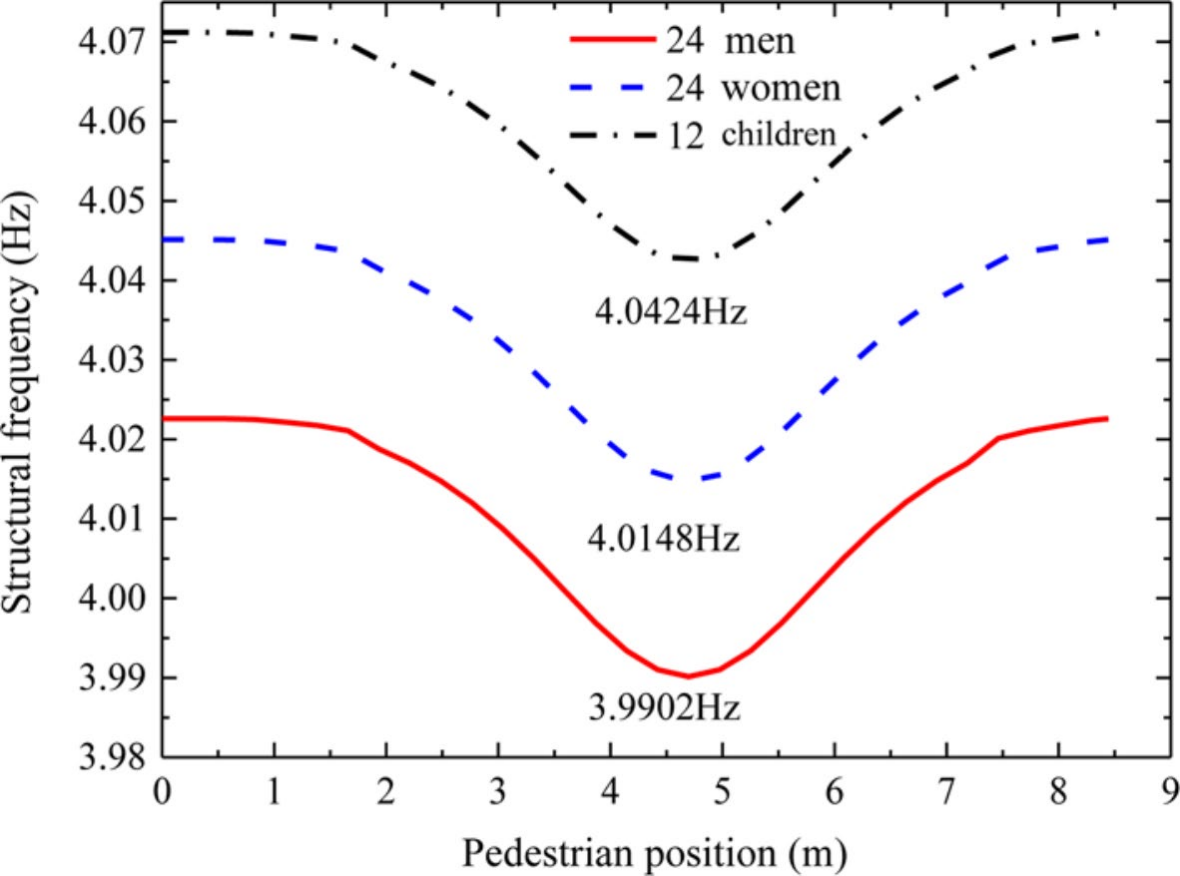
Mean value curve of floor frequency of influence of different human dynamic parameters.

**Fig. 3:**
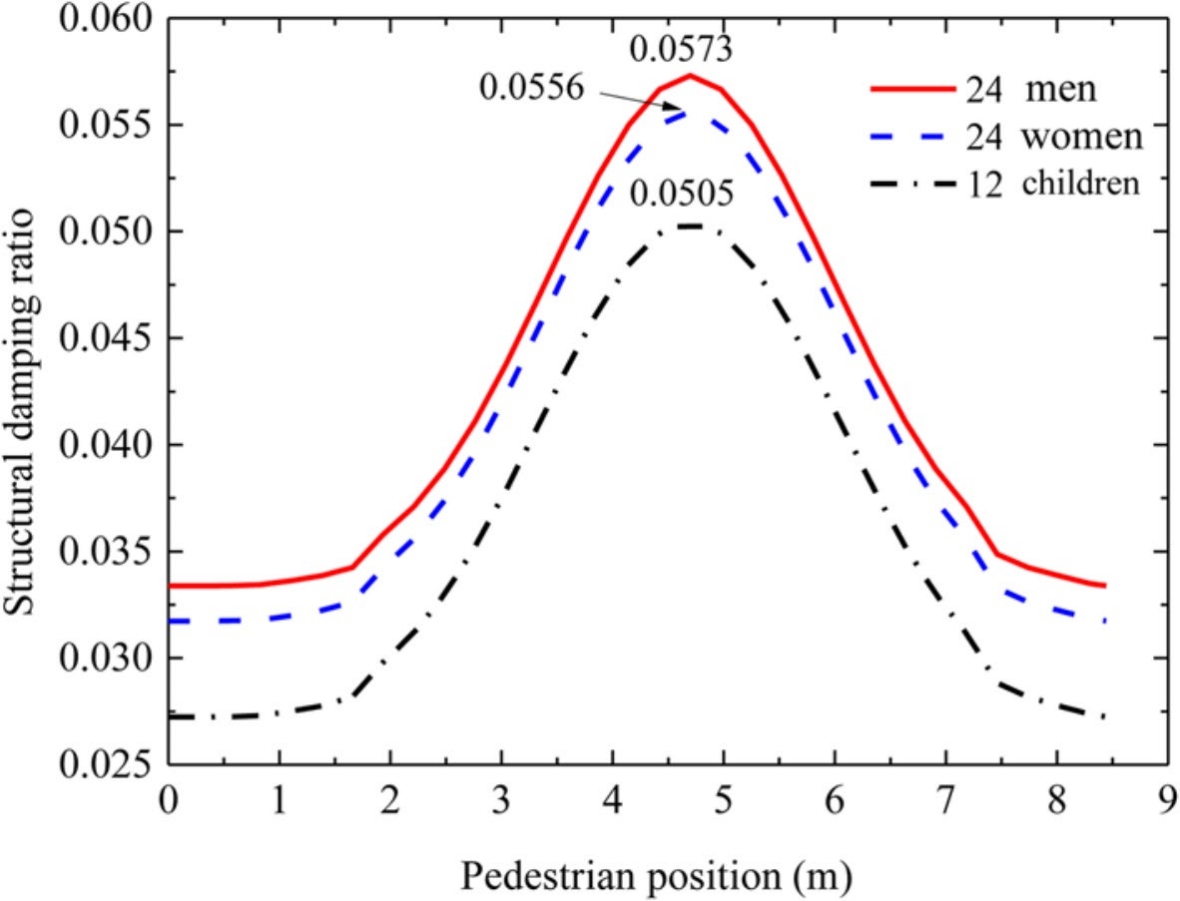
Mean value curve of floor damping of influence of different human dynamic parameters.

**Fig. 4:**
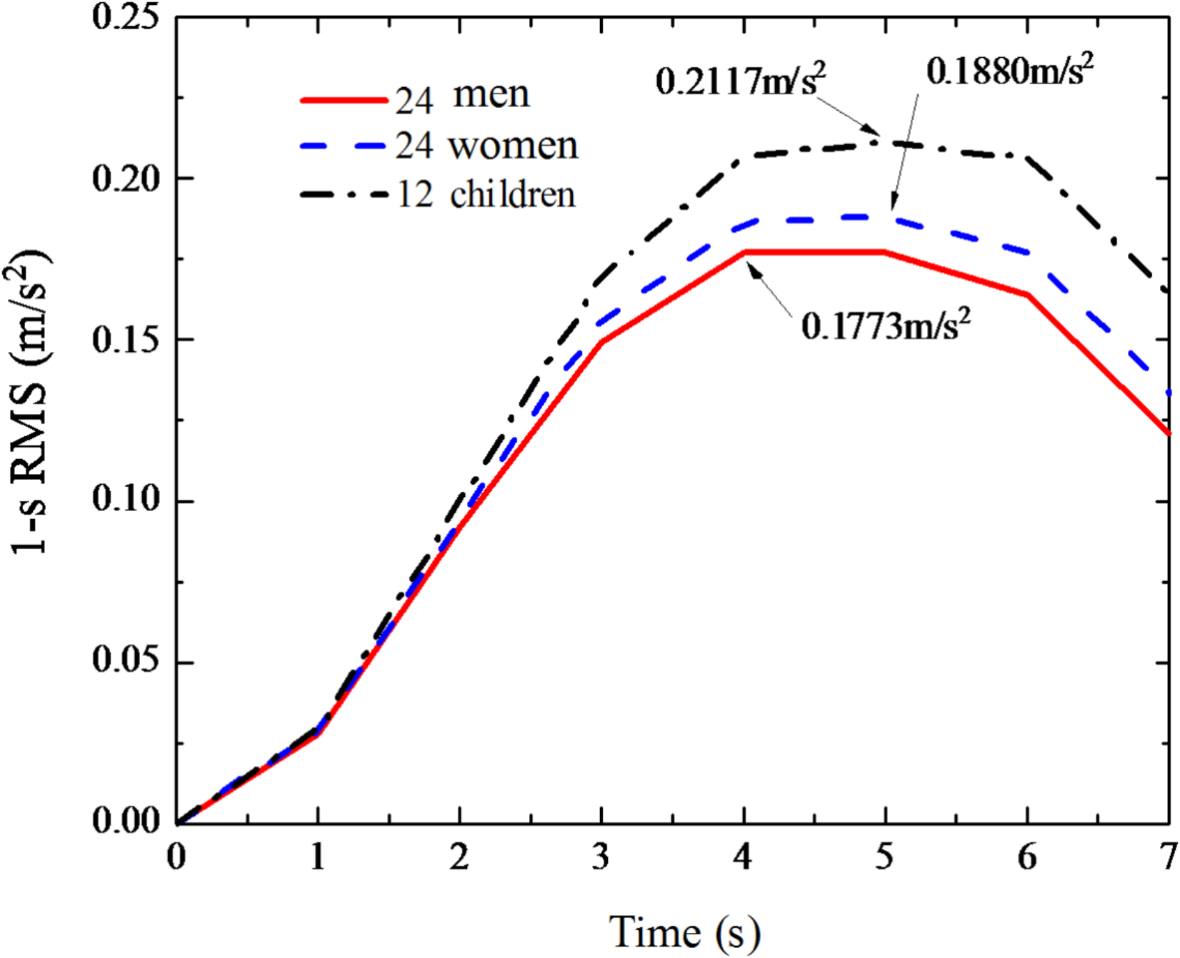
1-s RMS mean value curve of floor acceleration.

**Fig. 5:**
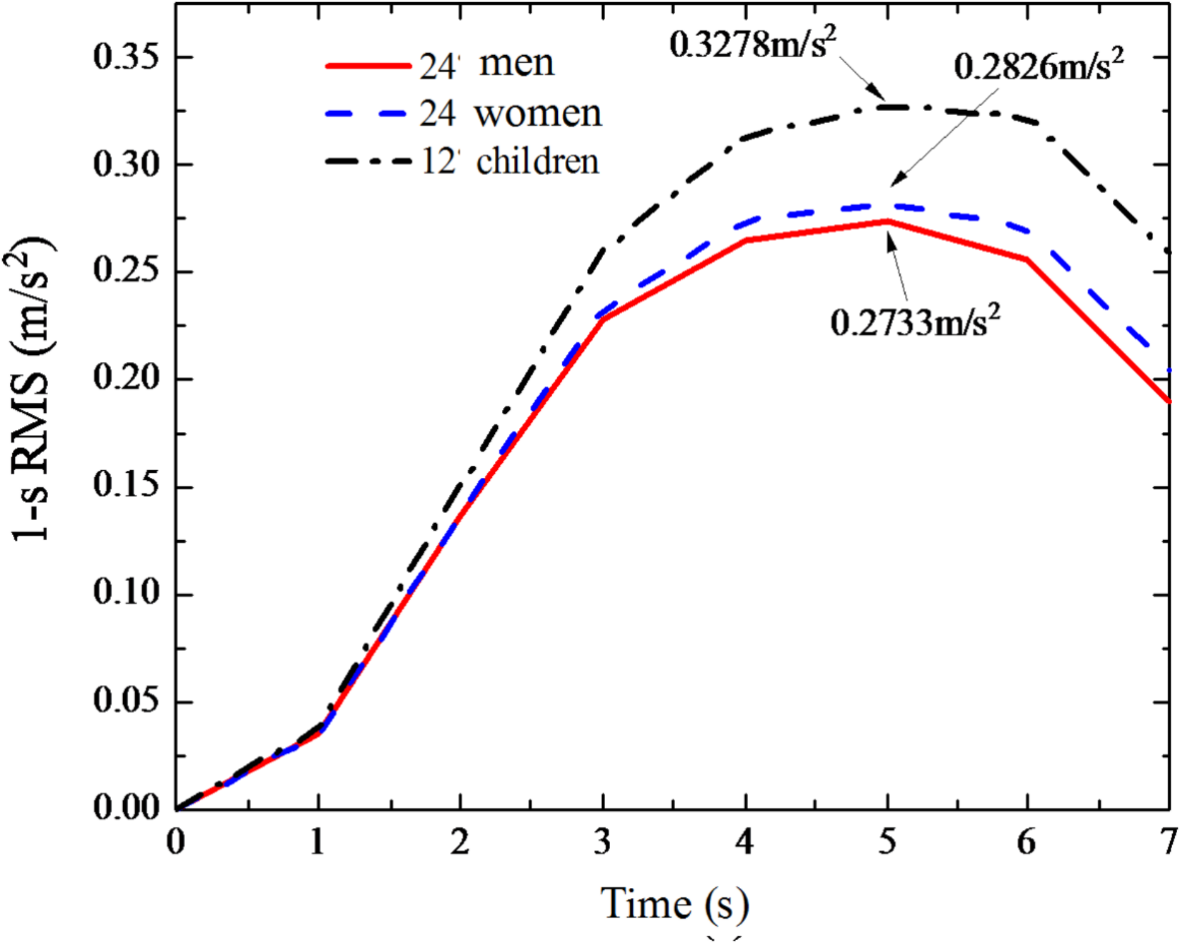
1-s RMS mean value curve of perceptive acceleration.

Through calculation, the first three vertical natural frequencies of the floor with no loads are 4.1452 Hz, 4.7030 Hz and 6.5958 Hz respectively. In order to study the influence of different human body parameters on the structural dynamic parameters, this section takes the pedestrian’s step frequency and mass as fixed values, and the human body parameters of the perceivers as variables. When the pedestrian step frequency passes through the floor at one-third of the structural fundamental frequency (1.3817 Hz, resonant frequency), 60 men, women and children with different dynamic parameters were selected to sit in the middle of the floor span, and the changes of structural dynamic parameters with time in the pedestrian-structure-sensing coupling system are calculated respectively. The 60 perceivers were made up of 24 men, 24 women and 12 children. In Figs. 2, 3, 4 and 5, the data for the mean calculation results of the 12 children, 24 women, and 24 men as research subjects are represented by black dashed lines, blue dotted lines, and red solid lines, respectively.

Figure 2 shows the mean value curve of floor frequency of the influence of different human dynamic parameters. As can be seen from Fig. 2, when 24 males, 24 females and 12 children are seated and standing respectively, with the change of pedestrian position, when pedestrians walk to the middle of the floor span, the impact on the floor frequency is the most significant and the frequency value reaches the minimum, which are 3.9902 Hz, 4.0148 Hz and 4.0424 Hz respectively. In compared with no-load the floor frequency of 4.1452 Hz decreased by 3.74%, 3.15% and 2.47%, respectively. In comparison, it can be seen that men have a greater impact on the floor frequency, followed by women and children. By comparing male and female, the influence of gender on floor frequency is also different, with a difference of 0.59%. Compared with adults (male and female) and children, the influence of human dynamic parameters at different ages on floor frequency is also different.

Figure 3 shows the mean value curve of floor damping ratio of the influence of different human body dynamics. It can be seen from the analysis of Fig. 3 that when the pedestrian walks to the mid span position of the floor, the damping effect on the floor is most significant, and its value reaches the maximum. For 24 men, 24 women and 12 children, the average structural damping ratio was 0.0573, 0.0556 and 0.0505 respectively, and the floor damping ratio 0.02 increased by 186.50%, 178.00% and 152.50% when compared with no-load. It can be seen that men have a greater impact on floor damping, followed by women and children. Similarly, for different gender perception, the impact on floor damping is different, with 8.50% difference between men and women. Perceptors of different ages have different effects on floor damping, with 34.00% and 25.50% differences between men and women and children respectively.

It can be seen from Figs. 2 and 3 that, with the change of pedestrian walking position, the frequency of the structure reaches the minimum and the structural damping ratio reaches the maximum when the pedestrian walks near the mid span position of the structure in the pedestrian structure sensing coupling system. The influence of male self dynamic parameters on the structure frequency and damping is greater than that of female and child self dynamic parameters.

### Influence of different human dynamic parameters on structural dynamic response

In order to study the influence of different human dynamic parameters on the structural dynamic response, this section also takes the step frequency and mass of pedestrians as fixed values, and the human dynamic parameters of the perceivers as variables. When different perceptors sit in the middle of the floor span, the same pedestrian step frequency passes through the floor with one third of the structural fundamental frequency (1.3817 Hz). The dynamic responses of the floor and the perceptors are calculated respectively, expressed in the form of 1-s root mean square (RMS) acceleration response, and the 1-s RMS generated by the corresponding floor of 24 men, 24 women, and 12 children is averaged. Figure 4 shows the 1-s RMS mean value curve of floor acceleration. It can be seen from Fig. 4 that in the pedestrian structure perception coupling system, when 60 perceptors sit in turn in the middle of the floor span, the peak values of 1-s RMS mean values generated by the corresponding floor of 24 men, 24 women and 12 children are 0.1773 m/s^2^, 0.1880 m/s^2^ and 0.2117 m/s^2^ respectively, showing the phenomenon that children are the largest, women are the second largest and men are the smallest. Comparing the effects of different genders (24 men and 24 women) on floor response, the mean peak value of floor RMS for females increased by 6.03% compared with that for males; comparing the effects of different ages (24 men, 24 women and 12 children) on floor response, The mean peak value of 1-s RMS of sitting and standing children increased by 19.40% and 12.60% compared with that of male and female children, respectively.

Figure 5 shows the 1-s RMS mean curve of the perceiver acceleration. In the pedestrian-structure-perception coupling system, taking the lightweight CFS composite floor as an example, pedestrians walk on the floor at a step frequency of one-third of the structural fundamental frequency (1.3817 Hz).The responses of the floor and the perceiver are calculated respectively, and the 1-s RMS curves of each are obtained. The calculations yielded the following results: The mean peaks of 1-s RMS of self-dynamic responses of 24 males, 24 females and 12 children were 0.2733 m/s^2^, 0.2826 m/s^2^ and 0.3278 m/s^2^, respectively. For perceivers of different genders, the peak value of male and female increased by 3.40%. For different ages, the peak value of children increased by 19.94% and 15.99%, respectively, compared with that of males and females. Compared with the peak value of the floor in Fig. 4, the peak value of the perceiver’s acceleration is greater than that of the floor. From the calculation results, it can be seen that in practical engineering applications, the dynamic response of the perceiver in the coupled system should be considered as greater than that of the floor slab to avoid the comfort index of the perceiver exceeding the limit due to the influence of different human dynamic parameters on the CFS composite floor.

Therefore, in the actual engineering practice, during the design of the CFS structural system and the construction of the comfort evaluation method, the response of the perceiver should be taken into account to better meet the comfort requirements of the floor slab.

### Influence of different pedestrian walking frequencies on structural dynamic parameters

In order to study the influence of step frequency of different pedestrian on the structural dynamic parameters, the step frequency of pedestrian was taken as a variable and the human dynamic parameters of the perceiver were taken as a fixed value. In the pedestrian-structure-perception coupling system, pedestrians pass through the floor at a slow pace frequency, and the average value of 60 perceptors’ dynamic parameters is selected as the perceptor’s dynamic parameters, as shown in Table 1 of literatures^27^. According to Sect. 3.1, the structural frequency and damping ratio are the minimum and the maximum when the pedestrian walks to the span of the structure. Therefore, the frequency and damping ratio of the floor when the pedestrian walks to the span of the structure at different step frequencies are calculated and extracted.

**Fig. 6:**
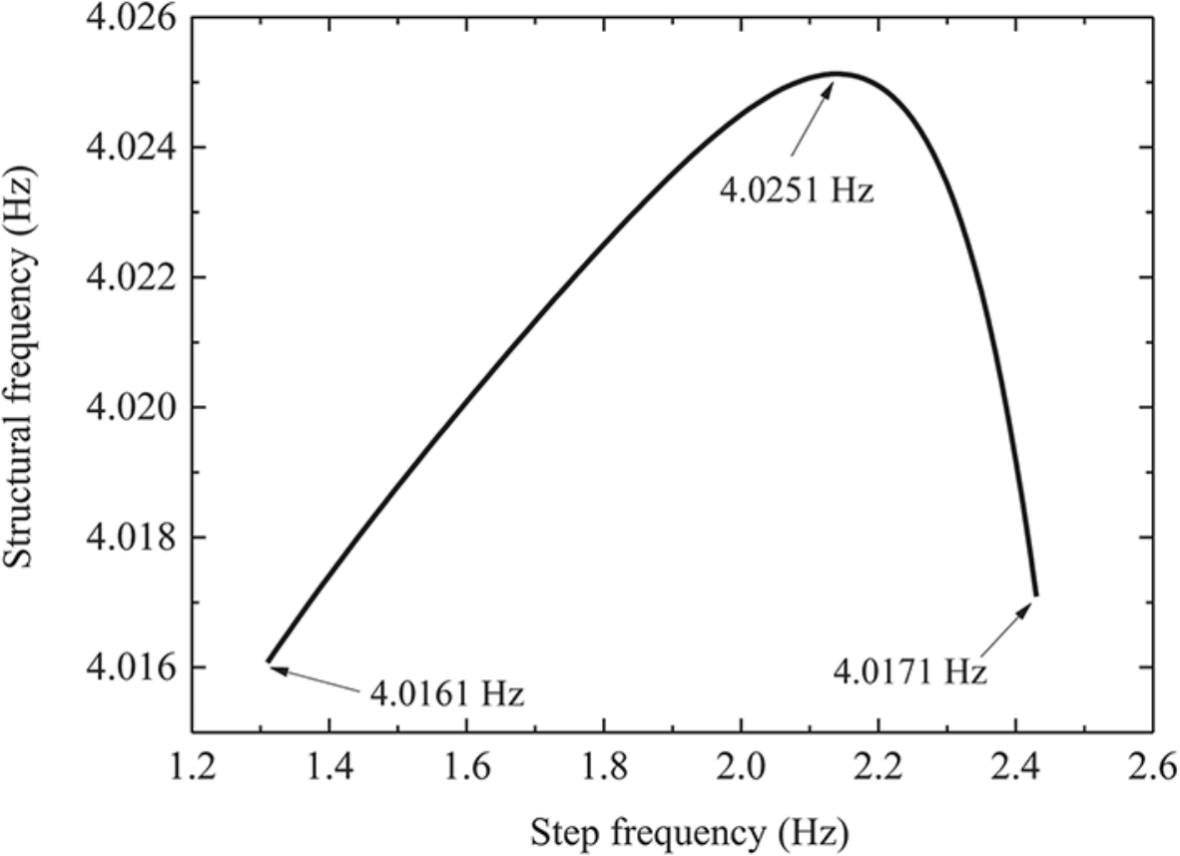
Frequency variation curve of floor when pedestrian passes through the middle span of the floor at a slow pace.

**Fig. 7:**
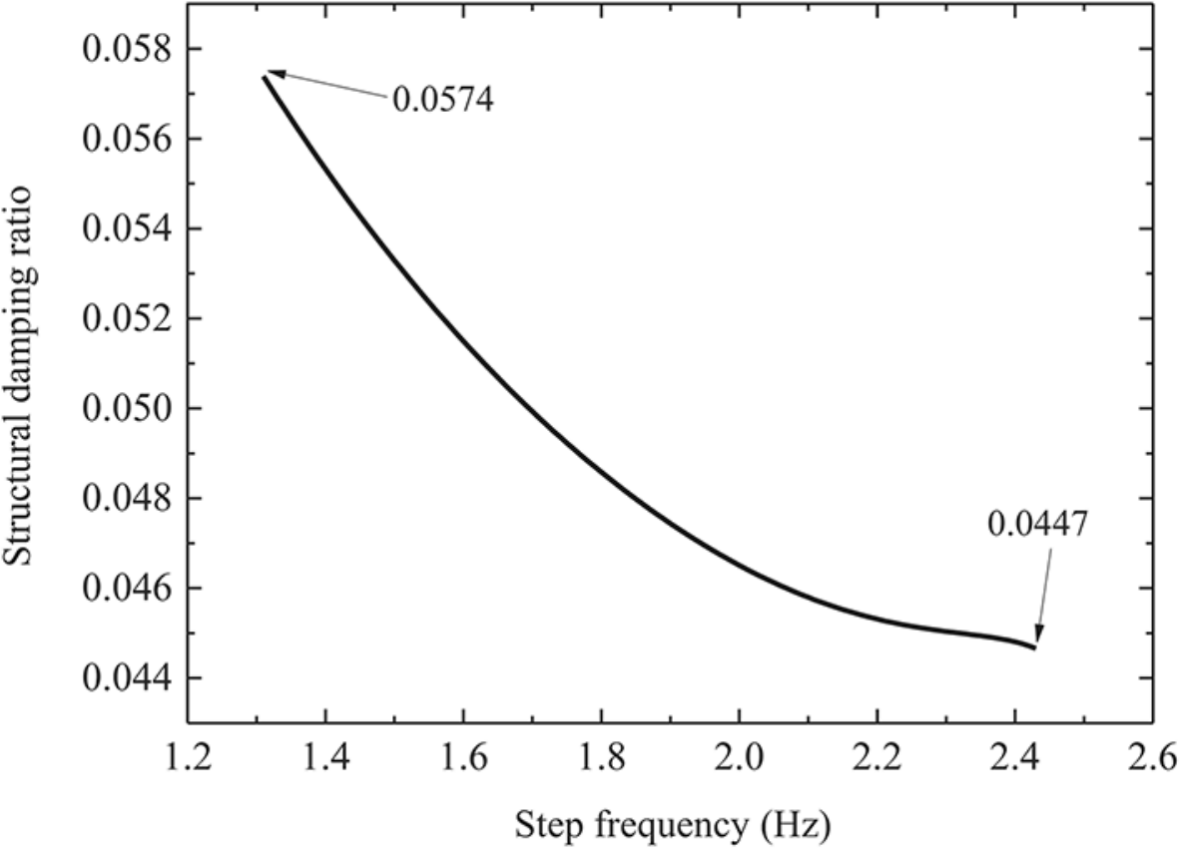
Damping ratio variation curve of floor when pedestrian passes through the middle span of the floor at a slow pace.

**Fig. 8:**
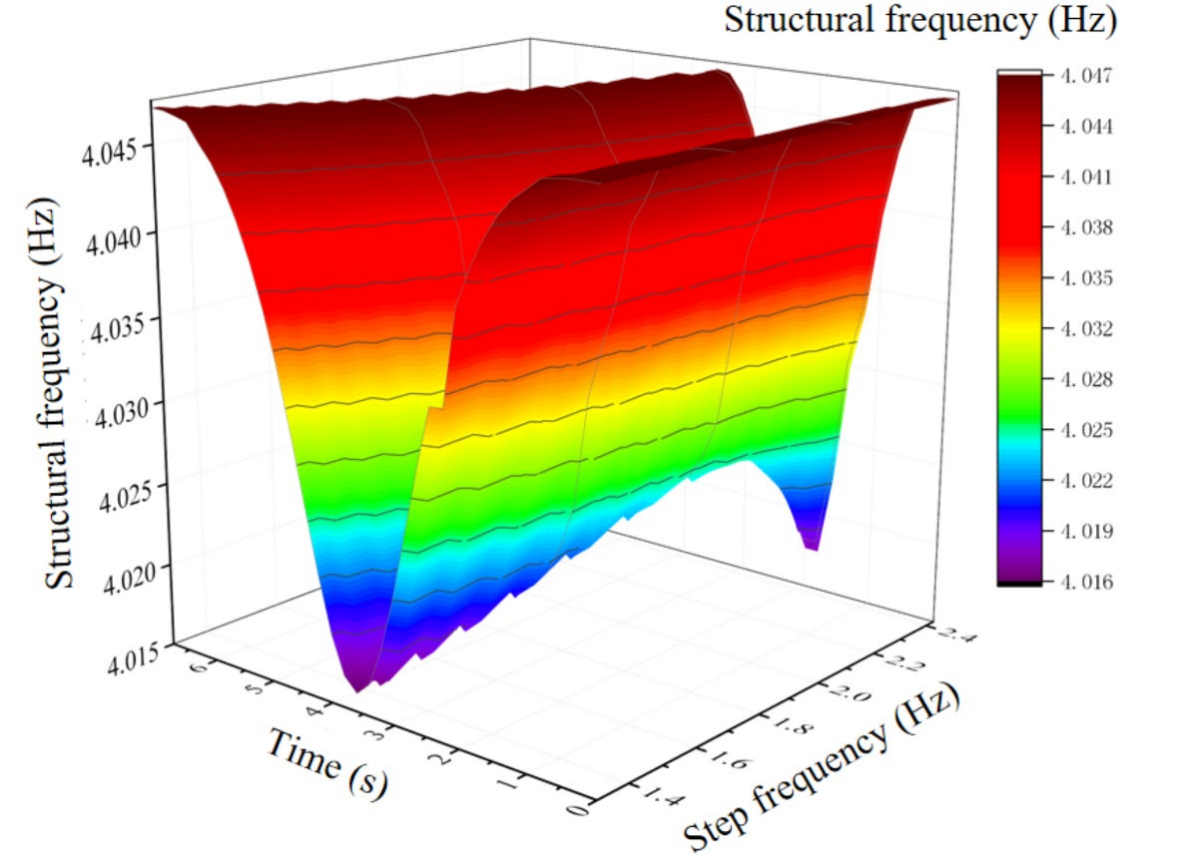
Effect of each step frequency on floor frequency in slow speed.

**Fig. 9:**
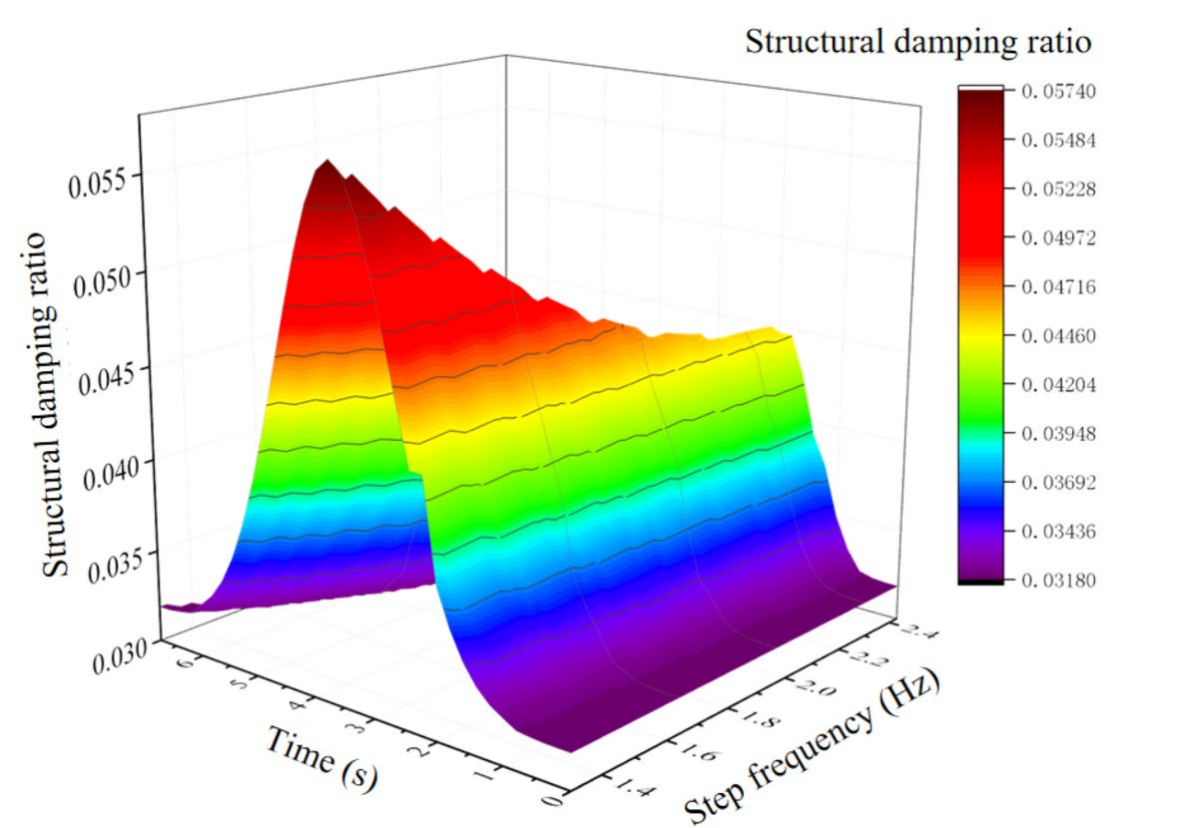
Effect of each step frequency on floor damping ratio in slow speed.

Figure 6 shows the variation curve of floor frequency when the pedestrian walks to the floor span at a slow pace. It can be seen from the Fig. 6 that, with the increasing of step frequency, the floor frequency presents a trend of first increasing and then decreasing, but it is smaller than the fundamental frequency when the floor is unloaded. When the step frequency is 1.31 Hz, the floor frequency change is the largest, which is 4.0161 Hz. Compared with the floor with no load, the fundamental frequency decreases by 3.11%. When the floor frequency changes to 4.0251 Hz, the step frequency at this time is 2.14 Hz, which is near half of the fundamental frequency of the structure (2.0726 Hz), namely, the structure appears resonance phenomenon at this time, indicating that when the pedestrian step frequency is near the double frequency of the fundamental frequency of the structure, the structure will cause resonance. Figure 7 shows the variation curve of floor damping ratio when the pedestrian walks to the floor span at a slow pace. With the increasing of step frequency, the floor damping ratio shows a decreasing trend. Figures 8 and 9 show the influence of each step frequency on floor frequency and damping ratio at a slow speed respectively, and the variation rule is consistent with Figs. 6 and 7.

By systematically analyzing and comparing the existing research on the comfort assessment of CFS composite floor structures^28–30^ and pedestrian load studies^31–33^, and in combination with the aforementioned data analysis, it is suggested that pedestrians should walk slowly in office environments to avoid a decrease in comfort levels due to increased walking speed. Additionally, based on the analysis of the aforementioned case, it is necessary to consider incorporating the peak acceleration of perceivers as a basis for comfort assessment when evaluating human-induced vibrations. This means adopting a full-path floor vibration comfort assessment method that encompasses excitation, propagation, and perception.

## Conclusion

In this paper, the effects of different human dynamic parameters on the vertical dynamic characteristics of the pedestrian-structure-sensing coupling system are studied. The deficiency is the lack of experimental data support, which will be used as a supplement for subsequent research. This paper mainly takes the CFS composite floor with four hinged sides as an example to calculate, and can also be extended to other floor with boundary conditions to provide some reference. The conclusions of this paper are as follows:

- Taking the office environment as an example, considering the dynamic coupling interaction among the perceiver, pedestrian, and structure, the response of multiple individuals should be taken into account in the existing evaluation of floor comfort and pedestrian load research. In the design and comfort evaluation of CFS composite floors, it is also necessary to statistically analyze the peak accelerations of different types of perceivers to establish a comprehensive comfort index for perceivers.
- With the change of pedestrian position, in the pedestrian-structure-perception coupling system, the floor frequency gradually decreases from end to mid-span, while the structural damping ratio gradually increases from end to mid-span. The influence of male dynamic parameters on the structural frequency and damping is greater than that of female and children. Their values were 3.74% and 186.50%, respectively.
- The different human dynamic parameters of the perceiver also have different influences on the dynamic response of the floor, showing that children are the largest, followed by women and men are the least. By comparing the influence of different genders on floor response, the mean peak value of floor RMS for female is 6.03% higher than that for male. By comparing the influence of different ages on floor response, the mean peak value of floor RMS for children is 19.40% higher than that for male and 12.60% higher than that for female.
- When the pedestrian step frequency is near the double frequency of the fundamental frequency of the structure, the structure resonance occurs, but the influence on the structure frequency is smaller than that of other step frequencies. With the increasing of step frequency, the damping ratio of the floor shows a decreasing trend, and the damping ratio of the structure increases by 187.00% and 123.50% when the damping ratio is at the highest and lowest point, respectively.

## Data Availability

All data generated or analysed during this study are included in this published article.
